# Supplementary material for: Census Demographics and Chlorpyrifos Use in California’s Central Valley, 2011–15: A Distributional Environmental Justice Analysis
Source: Int J Environ Res Public Health. 2020 Apr 10;17(7):2593. doi: 10.3390/ijerph17072593 (PMC7177971; doi:10.3390/ijerph17072593)
Supplement: Supplementary file 1 [file ijerph-17-02593-s001.pdf]

Supplemental Material for 'Census Demographics and Chlorpyrifos Use in California's Central Valley, 2011-15: A Distributional Environmental Justice Analysis'

Daniel J. Hicks

Contents

1 Spatial Weights **1**

2 Regression Specification **2**

3 Model Selection and Evaluation **3**

4 IV Impacts **3**

References **6**

1 Spatial Weights

To analyze the effect of choice of spatial weights, 8 row-normalized spatial weight constructions were considered for both tracts and places: contiguity, inverse distance weights (with an outer limit of 50 km and a decay of $\frac{1}{d}$), and k-nearest-neighbors (KNN) with k ranging from 3 to 8. As noted above, 62% of places had no contiguity-based neighbors. Moran's I was calculated for population densities corresponding to independent variables, e.g., density of Hispanic population, calculated as the number of Hispanic residents per square kilometer.

Among tracts, contiguity produced the highest values of Moran's I, followed by KNN, and finally distance weights. For example, for Hispanic population density, Moran's I was slightly less than .7 for contiguity weights; was between .45-.55 for KNN; and was slightly greater than .3 for distance weights. Among places, KNN and distance-based weights were similar, especially for larger values of K, while contiguity-based values were much smaller for most variables. For example

for Hispanic population density, Moran's I was between .55-.65 for KNN; about .53 for distance weights; but only .3 for contiguity weights.

LeSage and Pace (2014) argue that inferences in spatial statistical analysis are much less sensitive to the choice of spatial weights than is typically thought. By contrast, in the context of the present study, the choice of CTD produces multiple-order-of-magnitude differences across DV values. I therefore judged that different CTD values were likely to be a more important source of variation in effects than different spatial weights. KNN weights produced moderate and consistent values of Moran's I across tracts and places, and therefore KNN weights with $k = 3$ were selected for use in further analysis.

2 Regression Specification

For higher CTD values (30, 60, 90), distributions are bi- or trimodal. Plotting separate distributions for each county suggested that this was due to very different county-level baselines. For counties with many tracts or places, the distribution of values appeared to be sufficiently Gaussian for standard regression. County-level dummy variables were considered, and were found to improve homoscedasticity in non-spatial models, but also introduced multicollinearity in spatial lagged models (see below). However, inspection of residuals in the spatial Durbin models suggested that lagged dependent variables substantially improved homoscedasticity without county-level dummies. Still, even the spatial Durbin models exhibit some heteroscedasticity. Separate county-level regression models were therefore constructed. These models could only be fit for counties with 30 or more places or 50 or more tracts, which excluded many counties; and many of the estimates had high uncertainty. Full-data models are therefore used for primary analysis, with county-level models used as a robustness/heterogeneity check.

Spatial exploratory data analysis of both independent and dependent variables suggested substantial degrees of spatial autocorrelation on both sides of the regression formula (tables **??** and **??**). A sequence of three model specifications was considered: "standard" linear regression, without any spatial component (referred to simply as "regression" below); spatial regression with lagged independent variables, or "spatial lag X"; and spatial Durbin regression, which incorporates lags for both dependent and independent variables (LeSage and Pace 2009). If the regression model is specified as

$$Y = \alpha 1_n + X\beta + \varepsilon \tag{1}$$

where α is a scalar parameter, 1_n is a length-n column vector of 1s, X is a $n \times p$ design matrix, β is a length-p column vector of regression coefficients, and ε is a Gaussian noise term, then the spatial lag X model is specified as

$$Y = \alpha 1_n + X\beta + WX\theta + \varepsilon \tag{2}$$

where W is the $n \times n$ spatial weights matrix and θ is a length-p column vector of coefficients. The spatial Durbin model is further specified as

$$Y = \alpha 1_n + X\beta + WX\theta + \rho WY + \varepsilon \tag{3}$$

$$Y = (I_n - \rho W)^{-1}(\alpha 1_n + X\beta + WX\theta) \tag{4}$$

where ρ is a scalar parameter and I_n is the $n \times n$ identity matrix.

3 Model Selection and Evaluation

Model selection considered a "standard" non-spatial linear regression, spatial lagged X, and spatial Durbin models across each of the $10 = 2 \times 5$ geography-CTD combinations. This can be interpreted as taking the "general-to-specific" approach to spatial model selection (Elhorst 2010).

KNN spatial weights, with $k = 3$, were used for all of these models. This sequence of models was fitted for both places and tracts and for CTD values 10 through 90. Residual plots were examined for indications of heteroscedasticity, and R^2, AIC, and Moran's I of the residuals were compared within model-dataset combinations.

Across each of the 10 geography-CTD combinations, spatial Durbin models consistently outperformed both regression and spatial lagged X models in terms of AIC, Moran's I, and visual inspection for heteroscedasticity. Spatial Durbin models were therefore selected for further analysis.

However, Moran's I was still substantially greater than 0 for all spatial Durbin models, with values of approximately .07 for tracts and .15 for places. This suggests that there may be spatial non-stationarity; that is, the effects of the independent variables may vary across different sub-regions in the study area.

County-level regression models were used to examine this possibility further. (No resampling was done for county-level models.) Moran's I was consistently close to 0 ($\pm.05$) for some county-CTD combinations; but was still greater than .05 for tracts in several counties, especially with larger CTD values. In contrast, Moran's I was consistently substantially negative (less than $-.05$) for places in Fresno and Kern county. Thus, even at the county level, there are indications of spatial non-stationarity. Non-stationary models were not explored in the current study, but may be an important direction for future work.

4 IV Impacts

Unlike non-spatial linear regression models, spatial models treat observations — locations or geographic units — as statistically dependent. This means that changes in an IV at one location can influence the DV at another location,

corresponding to the term $WX\theta$. Further, the spatial Durbin model's lagged dependent variable term, $WY\rho$, introduces the possibility for feedback loops: a change in IV Δx_i at location l induces a change $\Delta y_{l'} = w_{l'l}\theta\Delta x_i$ in y at neighbor l', which feeds back to location l as $w_{ll'}\Delta y_{l'}\rho$. (This and the next paragraph generally follow LeSage and Pace (2009), §2.7.)

Spatial econometricians have introduced the notion of *impacts* for the interpretation of regression coefficients under spatial feedback. In non-spatial linear regression models without interaction, the coefficient β_i for IV x_i is identical to the partial derivative $\partial y/\partial x_i$. β_i can therefore be interpreted directly as the marginal effect of x_i on y (bracketing concerns about causal inference, etc.). But in the spatial Durbin model, the partial derivative

$$\frac{\partial y}{\partial x_i} = (I_n - W\rho)^{-1}(I_n\beta_i + W\theta_i) = S_i(W)$$

depends not just on the coefficients β_i and θ_i, but also the autoregression coefficient ρ. And the value of this partial derivative at location l depends on its connection to other locations, as encoded in W. The *total impacts* for IV x_i are formally defined as the mean row sum of $S_i(W)$, which corresponds to averaging $S_i(W)$ across locations.

Figures

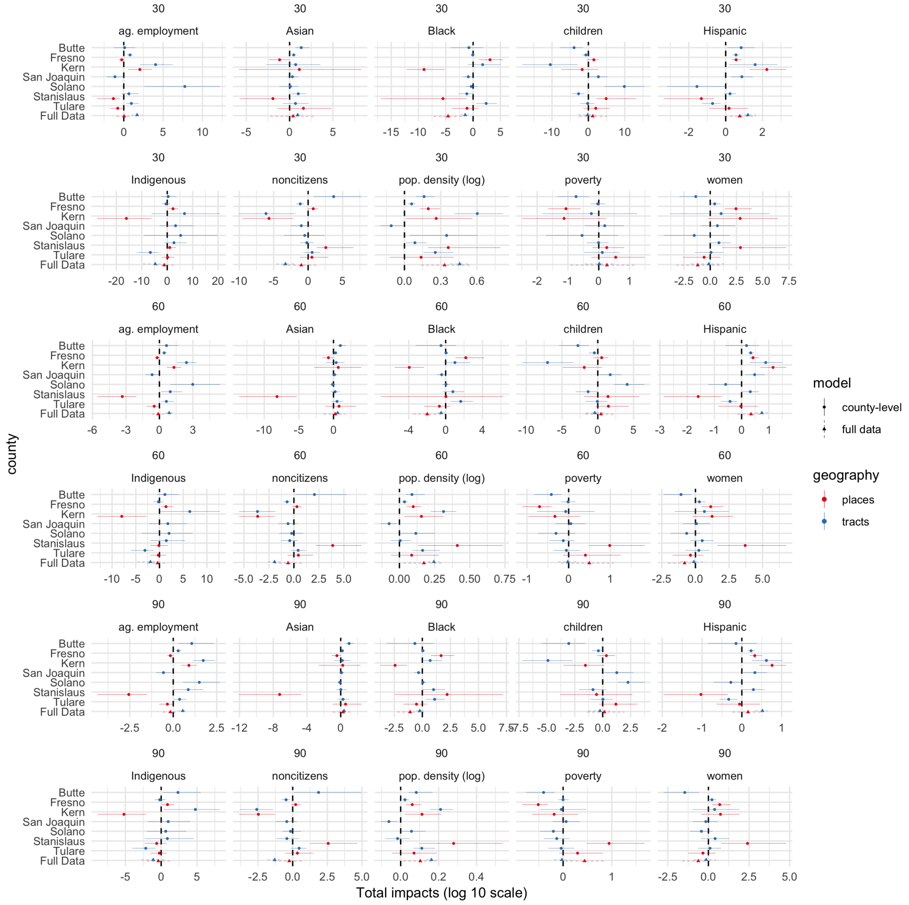

Figure S1: Total impacts from county-level models, with non-resampled full data estimates for comparison. All estimates on log scale. Tract estimates in blue; place estimates in red. Ends of line ranges indicate 5th and 95th percentiles of Monte Carlo impact draws; circles/triangles indicate medians.

References

Elhorst, J. Paul. 2010. “Applied Spatial Econometrics: Raising the Bar.” *Spatial Economic Analysis* 5 (1): 9–28. https://doi.org/10.1080/17421770903541772.

LeSage, James, and R Kelley Pace. 2009. *Introduction to Spatial Econometrics*. Boca Raton, FL: Chapman & Hall/CRC.

LeSage, James P., and R. Kelley Pace. 2014. “The Biggest Myth in Spatial Econometrics.” *Econometrics* 2 (4): 217–49. https://doi.org/10.3390/econometrics2040217.
